# Targeting redox biology to reverse mitochondrial dysfunction

**DOI:** 10.18632/aging.100590

**Published:** 2013-08-10

**Authors:** David J. Marcinek, Michael P. Siegel

**Affiliations:** ^1^ Departments of Radiology, Bioengineering, and Pathology. University of Washington Medical School, Seattle, WA 98195, USA; ^2^ Deparment of Biomedical Engineering, Oregon Health & Science University, Portland, OR 97239, USA

There is general agreement that mitochondria play an important role in the aging process, but the role of mitochondrial oxidative stress remains controversial. Most previous work looking at mitochondrial oxidative stress has focused on damage to DNA, proteins, and lipids with age or in response to manipulation of cellular antioxidants. The interaction between oxidative damage and aging has been called into question in recent years by studies demonstrating little effect on aging and lifespan in mice with genetically modified antioxidant systems [1]. A notable exception is the life extension and protection against multiple diseases in mice that express catalase in the mitochondria, which suggests that the cellular location and type of reactive oxygen species is an important factor [2].

There is growing appreciation for a more subtle role for oxidative stress in aging. This perspective recognizes the important role of reactive oxygen and nitrogen species in regulating adaptive responses to physiological stress (i.e. redox signaling) [3]. One characteristic of this redox signaling is that it typically involves low levels of relatively short-lived oxidative stress under normal physiological conditions. Another important feature of the redox stress hypothesis in the context of aging is that the signaling is dynamic and reversible. Mitochondria are likely targets for regulation by redox signaling, because they are a major source of reactive oxygen species in most cells. This is supported by reports demonstrating that mitochondrial enzyme activities, metabolic fluxes, and morphology are sensitive to the redox environment ex vivo and in cells.

Our laboratory is interested in whether redox inhibition of mitochondrial function contributes to age-related energy deficits in vivo in mouse and human skeletal muscle [4, 5]. Siegel et al. [5] demonstrated that a mild oxidative stress induced by paraquat reproduced some of the age-related changes to in vivo mitochondrial function in mouse skeletal muscle. These changes were present within 24 hours of a single low paraquat dose and returned to normal after three days. Interestingly, mitochondrial energetics in skeletal muscle from old mice was more sensitive to this mild oxidative stress. Several studies have demonstrated that the redox environment in aged tissues is more oxidized [3] in part due to the greater H_2_O_2_ production from aged mitochondria. Therefore, the results from the paraquat experiments suggest that reversible redox inhibition of in vivo mitochondrial energetics may contribute to the mitochondrial aging phenotype.

**Figure 1:**
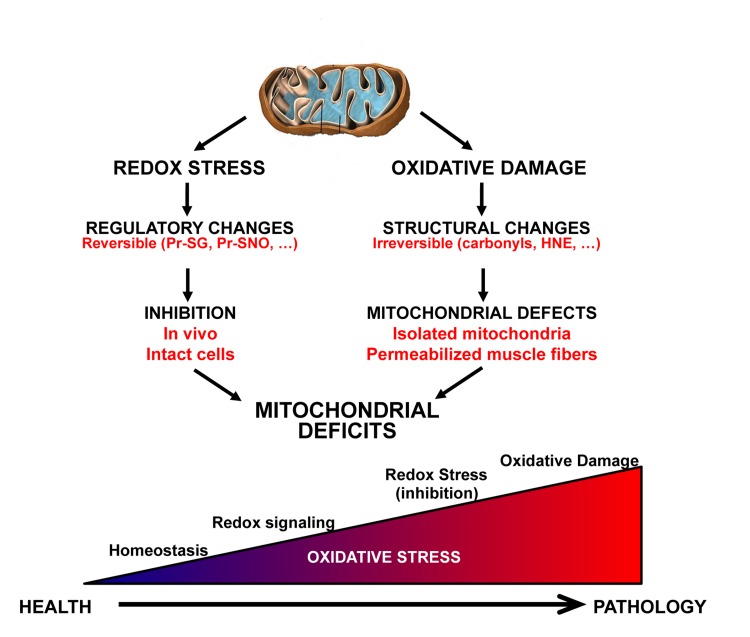
Illustration of key differences between the redox stress and oxidative damage mechanisms contributing to mitochondrial aging In the redox stress hypothesis an increase in oxidative stress leads to reversible regulatory changes in function that are dependent on the interaction between the mitochondria and the cell environment. In contrast, oxidative damage results in structural changes to macromolecules that are generally irreversible. The gradient on the bottom illustrates the continuum between healthy redox homeostasis and pathology.

Our recent article in Aging Cell [6] further tested this hypothesis using a mitochondrial targeted peptide, SS-31, known to reduce mitochondrial H_2_O_2_ production. SS-31 reduced the high mitochondrial H_2_O_2_ production from aged permeabilized muscle fibers from the extensor digitorum longus, but had no effect on young fibers. In the aged mice, one hour after in vivo treatment with SS-31 the cellular redox status measured by GSH:GSSG was more reduced. This was accompanied by improved mitochondrial coupling (P/O) and maximum ATP production in vivo in the skeletal muscle, while there was no effect on the mitochondrial energetics in young skeletal muscle. In addition to the improvements in muscle energetics, one hour and one week of SS-31 treatment resulted in improved muscle performance and increased exercise tolerance, respectively, in the old mice. This rapid reversal of in vivo energy deficits supports the hypothesis that mitochondrial deficits in aged skeletal muscle are, at least in part, due to reversible redox sensitive inhibition. Thus assessing the role of mitochondrial oxidative stress in aging and disease will require careful attention to changes to the in vivo redox environment and the mechanisms by which these changes can affect cell function.

The mechanisms linking mitochondrial redox changes, regulation of metabolic fluxes, and reversal of dysfunction by SS-31 remain unclear. One potential mechanism is the post-translation modification of mitochondrial protein thiols with age. There are several rapidly reversible redox sensitive modifications of protein thiol groups that have been shown to affect the activity of mitochondrial proteins. This has been a relatively understudied area in the context of aging. However, in light of our results with SS-31 this warrants further attention. SS-31 has also been shown to interact directly with cardiolipin and preserve mitochondrial morphology [7]. Given the dynamic nature of mitochondrial morphology, stabilization of cardiolipin or redox control of fission and fusion also may contribute to rapid improvements in mitochondrial function [8].

Expanding the mitochondrial oxidative stress theory of aging to include reversible redox signaling opens new avenues for the development of potential therapeutics. This shift identifies the mitochondrial and cellular redox environment as potential targets for therapeutic intervention to reverse tissue dysfunction in age. Perhaps even more clinically relevant is the potential to focus on short-term treatment to reverse dysfunction. Translation of this strategy to humans would allow clinicians to treat elderly patients after mitochondrial or significant muscle dysfunction is identified, thus bypassing the need for costly long-term preventative treatments.
